# Genomic mating as sustainable breeding for Chinese indigenous Ningxiang pigs

**DOI:** 10.1371/journal.pone.0236629

**Published:** 2020-08-14

**Authors:** Jun He, Xiao-Lin Wu, Qinghua Zeng, Hao Li, Haiming Ma, Juan Jiang, Guilherme J. M. Rosa, Daniel Gianola, Richard G. Tait Jr., Stewart Bauck

**Affiliations:** 1 College of Animal Science and Technology, Hunan Agricultural University, Changsha, Hunan, China; 2 Biostatistics and Bioinformatics, Neogen GeneSeek, Lincoln, NE, United States of America; 3 Department of Animal Sciences, University of Wisconsin, Madison, WI, United States of America; 4 Ningxiang Pig Farm of Dalong Livestock Technology Co., Ltd., Ningxiang, Hunan, China; University of Connecticut, UNITED STATES

## Abstract

An important economic reason for the loss of local breeds is that they tend to be less productive, and hence having less market value than commercial breeds. Nevertheless, local breeds often have irreplaceable values, genetically and sociologically. In the breeding programs with local breeds, it is crucial to balance the selection for genetic gain and the maintaining of genetic diversity. These two objectives are often conflicting, and finding the optimal point of the trade-off has been a challenge for breeders. Genomic selection (GS) provides a revolutionary tool for the genetic improvement of farm animals. At the same time, it can increase inbreeding and produce a more rapid depletion of genetic variability of the selected traits in future generations. Optimum-contribution selection (OCS) represents an approach to maximize genetic gain while constraining inbreeding within a targeted range. In the present study, 515 Ningxiang pigs were genotyped with the Illumina Porcine SNP60 array or the GeneSeek Genomic Profiler Porcine 50K array. The Ningxiang pigs were found to be highly inbred at the genomic level. Average locus-wise inbreeding coefficients were 0.41 and 0.37 for the two SNP arrays used, whereas genomic inbreeding coefficients based on runs of homozygosity were 0.24 and 0.25, respectively. Simulated phenotypic data were used to assess the utility of genomic OCS (GOCS) in comparison with GS without inbreeding control. GOCS was conducted under two scenarios, selecting sires only (GOCS_S) or selecting sires and dams (GOCS_SD), while kinships were constrained on selected parents. The genetic gain for average daily body weight gain (ADG) per generation was between 18.99 and 20.55 g with GOCS_S, and between 23.20 and 28.92 with GOCS_SD, and it varied from 25.38 to 48.38 g under GS without controlling inbreeding. While the rate of genetic gain per generation obtained using GS was substantially larger than that obtained by the two scenarios of genomic OCS in the beginning generations of selection, the difference in the genetic gain of ADG between GS and GOCS reduced quickly in latter generations. At generation ten, the difference in the realized rates of genetic gain between GS and GOCS_SD diminished and ended up with even a slightly higher genetic gain with GOCS_SD, due to the rapid loss of genetic variance with GS and fixation of causative genes. The rate of inbreeding was mostly maintained below 5% per generation with genomic OCS, whereas it increased to between 10.5% and 15.3% per generation with GS. Therefore, genomic OCS appears to be a sustainable strategy for the genetic improvement of local breeds such as Ningxiang pigs, but keeping mind that a variety of GOCS methods exist and the optimal forms remain to be exploited further.

## Introduction

The past century has witnessed the development of a few intensively selected commercial breeds (and lines) in farm animals and the challenge that many more local breeds had been lost or are endangered [1,2]. The latter situation imposes a high risk to the world’s animal production if these commercial breeds run into genetic problems due to inbreeding or correlated selection responses, or if changes in the production section require traits that are poorly developed in the commercial breeds because there will be no alternatives [1]. Economics is a significant driver for the loss of local breeds, as they tend to be less productive, and hence having less market value than commercial breeds [2]. Nevertheless, local breeds are irreplaceable, both genetically and sociologically. For example, local breeds often are better adapted to the local environment, and therefore they tend to be more resistant to high temperatures or diseases than commercial breeds. Maximizing genetic gain and maintaining genetic diversity are conflict objectives in breeding programs [3], and finding the optimal level of trade-off has been a challenge for breeders. This is especially so with a local breed of small population size, which has a higher probability for loss of genetic variability and appearance of inbreeding depression than commercial breeds of large populations. In reality, crossing breeding has been used as an alternative strategy for improving the productivity of local breeds, but it, at the same time, accelerates the risk of losing or endangering local breeds [3,4]. For a sustainable selection that aims at achieving long-term genetic gain, it is crucial to have balanced goals that can control inbreeding by increasing heterozygosity and meanwhile keep genetic diversity of a population.

Genomic selection (GS) has been a revolutionary method for the genetic improvement of farm animals [5,6]. GS can accelerate breeding by shortening generation intervals, and to higher selection accuracy, especially for traits of low heritability or difficult to observe. However, GS can elevate the inbreeding rate (ΔF) rapidly, and high-production animals tend to have high kinship coefficients between them. The loss of diversity limits long-term gain for the trait under selection, and it also jeopardizes future breeding for other traits. In contrast, optimum-contribution selection (OCS) provides a strategy that balances genetic gain and diversity. OCS allows obtaining desirable genetic gain while constraining the rate of inbreeding in the progeny by restricting relationships between selected parents. Thus, it can maintain sustainable, long-term genetic gains [7–9]. OCS operates on the theory of genetic contributions [10], which states that the maximum rate of genetic gain, given a constraint on parental relationships, is realized when an exact threshold-linear relationship exists between the Mendelian-sampling of ancestral animals and their genetic contributions to the descendants of a population. Initially developed with a pedigree-based relationship matrix, OCS can align ancestors to the threshold-linear relationship only approximately [9,11]. This is because the true Mendelian-sampling terms of the ancestors are unknown, and they are estimated from pedigree information based on expectations but not realizations [9,12,13]. For example, the expected relationship between two full-sibs is 0.5, but the realized relationship can deviate considerably from 0.5, depending on the segregation of the parental chromosomes. With the availability of informative SNPs covering the whole genome, one can trace the inheritance of chromosomal segments and estimate relationships between selection candidates more accurately. Therefore, genomic OCS (GOCS, or OCS with genomic information) can increase the accuracy of assessment of Mendelian-sampling terms by aligning ancestors closer to the exact threshold–linear relationships than OCS based on pedigree information only [14].

Optimum contribution selection consists of a variety of algorithms and methods. Generally speaking, OCS maximizes genetic gain under a predefined genomic inbreeding rate by calculating the optimal contribution of all selected candidates to the next generation through a constrained optimization. Like pedigree OCS, genomic OCS has been refined considerably to accommodate operational breeding constraints, such as restricting the number of individuals contributing to the next generation and imposing upper or lower limits on how much an individual contributes. Some other forms of genomic mating and relevant computation strategies are also worth mentioning hereafter. Meuwissen [15] proposed to manage these additional constraints with an iterative heuristic wrapped around the original solution that removes individuals with too low contribution and truncates contributions exceeding the maximum then repeatedly reoptimizing the remaining contributions [16]. Alternatively, the operational constraints can be modeled directly using semidefinite programming, which may provide slightly higher gains at the cost of a more complex problem formulation [17,18]. A different strategy is to maximize a weighted index that balances genetic gain and inbreeding [19,20]. Optimizing this simple index with general-purpose metaheuristics, such as a differential evolution algorithm [21], allows one to comfortably accommodate alternative or additional objectives, thus trading optimality of solutions for flexibility. For example, this allowed Kinghorn [22] to move from assigning individual contributions to identifying optimal mating pairs. More recently, Gebregiwergis et al. compared alternative forms of genomic-relationship matrices to controlling coancestry and thereby future inbreeding by optimum contribution selection [23]. Their results highlight the importance of using genomic-relationship matrices that focus on QTL regions for GEBV estimation when the number of QTL is small in GOCS.

In pig breeding, there were a limited number of simulation studies that applied traditional OCS, such as Gourdine et al. [24] and Dagnachew and Meuwissen [25]. Genomic OCS applied to local pig breeds remains to addressed adequately. For example, the Ningxiang pig breed is one of the four most famous Chinese indigenous swine breeds in China, which was domesticated more than 5,000 years ago. Ningxiang pigs are well known for their meat flavor as they have over 5% intramuscular fat, compared to approximately 2% of imported commercial pigs [26,27]. Furthermore, they have adapted well to roughage utilization and local environments, and have high resistance to diseases. However, the market value of Ningxiang pigs is relatively low because their growth rate and feed efficiency are low. Thus, the selection of Ningxiang pigs is expected to increase the growth rate by some extend while at the same time, maintain its genetic diversity and control the inbreeding risk within an acceptable range. Nevertheless, the current breeding programs in Ningxiang pigs have been heavily targeted on the conversation without much investment in genetic gain. The purposes of the present study were: (1) to estimate the inbreeding level of Ningxiang pigs based on genomic information, and (2) to evaluate the effectiveness of genomic OCS as a sustainable selection strategy for the genetic improvement of Ningxiang pigs as compared to GS without inbreeding control.

## Materials and methods

### Animals and genotypes

DNA was isolated from ear or hair samples, collected from two sets of Ningxiang pigs. The NXP set 1 consisted of 212 pigs, which were raised in the conservation region of Ningxiang pigs and genotyped with an Illumina Porcine SNP60 Version 2 array (68,528 SNPs). The NXP set 2 has 303 pigs, which were raised in Ningxiang Pig Farm of Dalong Livestock Technology Co., Ltd. and genotyped with a GeneSeek Genomic Profiler (GGP) Porcine 50K Version 1 array (50,915 SNPs). These two populations were historically genetically correlated but have diverged from each other in the past decades due to closed breeding within each population. The number of genotyped SNPs and their minor allele frequencies by chromosome (chromosomes 1–18 and X) based on the Sscrofa10.2 assembly for the two sets of pigs are listed in Table 1. During the data cleaning, unmapped SNPs and those on mitochondrial and Y chromosomes were excluded. Genotypes were phased, and missing genotypes imputed using the FImpute software [28]. All animal procedures were carried out in accordance with the guidelines established by the China Council on Animal Care and were performed at Ningxiang Pig Farm of Dalong Livestock Technology Co., Ltd. The protocols were approved by the Experimental Animal Manage Committee of Ningxiang Pig Farm of Dalong Livestock Technology Co., Ltd. The data in this manuscript was provided by Ningxiang Pig Farm of Dalong Livestock Technology Co., Ltd., and was analyzed by Hunan Agricultural University.

**Table 1:** Number of SNPs and minor allele frequencies of SNPs by chromosome in 515 Ningxiang pigs after data edits, each genotyped with either an Illumina Porcine SNP60 array or GeneSeek Genomic Profiler (GGP) Porcine 50K array^a^^,^^b^.

Set I (212 pigs); Illumina Porcine SNP60			Set II (303 pigs); GGP Porcine 50K		
Chromosome	N	MAF (SD)	Chromosome	N	MAF (SD)
1	5,927	0.118 (0.156)	1	3,782	0.132 (0.155)
2	4,140	0.122 (0.159)	2	2,679	0.136 (0.155)
3	3,574	0.113 (0.153)	3	2,366	0.127 (0.153)
4	3,803	0.118 (0.156)	4	2,451	0.122 (0.155)
5	3,009	0.108 (0.151)	5	1,956	0.119 (0.153)
6	4,357	0.128 (0.157)	6	2,779	0.131 (0.153)
7	3,942	0.127 (0.156)	7	2,364	0.143 (0.157)
8	3,532	0.126 (0.159)	8	2,357	0.134 (0.158)
9	3,852	0.119 (0.156)	9	2,562	0.129 (0.154)
10	2,873	0.119 (0.155)	10	1,358	0.136 (0.157)
11	2,344	0.125 (0.160)	11	1,505	0.139 (0.162)
12	2,511	0.128 (0.159)	12	1110	0.140 (0.158)
13	4,432	0.121 (0.157)	13	2,839	0.130 (0.154)
14	4,086	0.129 (0.159)	14	2,672	0.141 (0.159)
15	3,605	0.118 (0.155)	15	2,336	0.125 (0.155)
16	2,386	0.132 (0.159)	16	1,484	0.133 (0.155)
17	2,176	0.121 (0.154)	17	1,178	0.140 (0.159)
18	1,756	0.102 (0.150)	18	1,040	0.113 (0.149)
X	3,304	0.058 (0.124)	X	2,455	0.070 (0.133)
Total	65,609	0.117 (0.155)	Total	41,273	0.128 (0.154)

^a^ N = Number of SNPs by chromosomes;

^b^ MAF (SD) = Mean (Standard deviation) of minor allele frequencies of SNPs by chromosomes.

The kinship between two individuals *i* and *j*, denoted by *f*_*ij*_, is the probability that two alleles randomly chosen from both individuals were IBD. The inbreeding of an individual, say *i*, is the probability that two alleles carried by this individual at a given locus are IBD. Kinship coefficients were calculated with two methods: 1) locus-wise (LW-kinship) or 2) as the proportion of the genome included in runs of homozygosity (ROH-kinship), which are contiguous regions of the genome where an individual is homozygous across all sites. ROH arises when two copies of an ancestral haplotype are brought together in an individual. Hence, that haplotype would be autozygous, i.e., homozygous by descent. In the present study, ROH is defined through a genomic scan for a minimum of 20 SNPs with zero heterozygous genotypes and zero missing genotypes, also subject to a maximum marker spacing of 100Kb between two neighboring SNPs within ROH.

Following Toro et al. [29], we computed the LW-kinship coefficients as follows:

$$f_{ij(LW)}=\frac{1}{M}\sum_{k=1}^{M}\left[\frac{f_{{IIS}_{ijk}}-p_{k}^{2}-q_{k}^{2}}{2p_{k}q_{k}}\right]$$ (1)

where *M* is the number of SNPs on the GGP 50K SNP array, *p*_*k*_ and *q*_*k*_ are the frequencies of the two alleles at locus *k* at the current generation, and $f_{{IIS}_{ijk}}$ is the probability that the two alleles are identical-in-state (IIS). Exactly, the allele frequencies need to be estimated from the base population. The latter, however, is unknown, and the population at generation 0 in the present study was not truly the base population.

ROH-kinship coefficients were computed based on segments of identity-by-status shared between pairs of individuals. These segments are potentially ROHs in the offspring of each mating pair [30]. That is,

$$f_{ij(ROH)}=\frac{1}{4T}\sum_{k=1}^{M}\sum_{a_{i}=1}^{2}\sum_{b_{j}=1}^{2}L_{k}(a_{i},b_{j})$$ (2)

where *L*_*k*_(*a*_*i*_,*b*_*j*_) is the length of the *k*-th shared IBD segment measured over homologous *a* of individual *i* and homologous *b* of individual *j*, and *T* is the total length of the genome.

Similar to the computing of kinship coefficients, the inbreeding coefficients were also computed locus-wise and based on ROH, respectively. The inbreeding coefficient of an individual can be viewed as the self ancestry with itself, which equal to $\frac{1}{2}(1+f_{ii})$. Precisely speaking, inbreeding is not directly the self coancestry, but it can be calculated from it as shown above. Then, the rate of inbreeding per generation was calculated in retrospect from the average inbreeding in the current generation (*F*_*t*+1_) deviated from that in the previous generation (*F*_*t*_), expressed relative to what remained to be non-inbred at generation *t* [31]:

$$\Delta F=\frac{F_{t+1}-F_{t}}{\left(1-F_{t}\right)}$$ (3)

### Simulation of phenotypes

**Fig 1:**
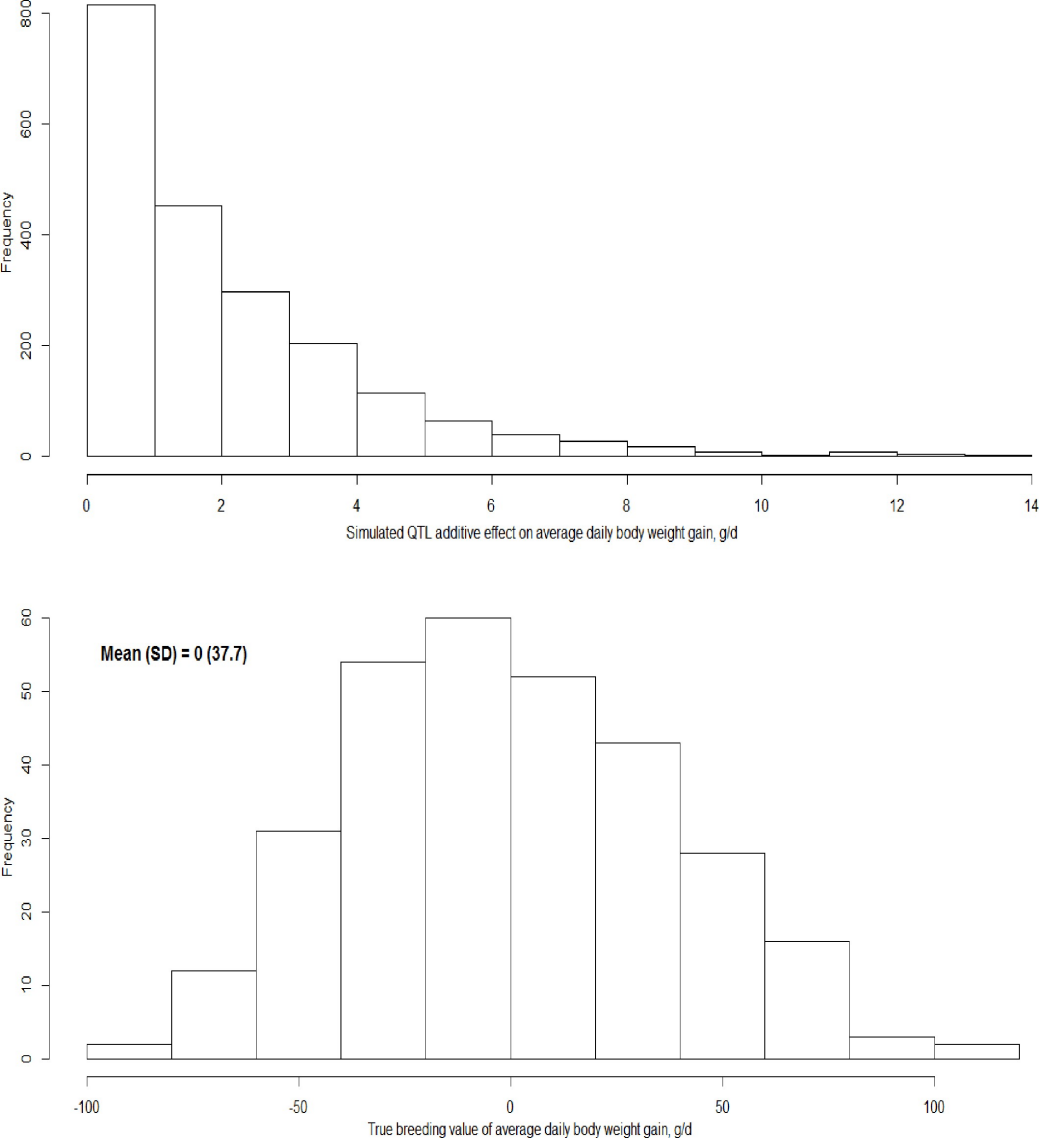
Distributions of simulated QTL effects (A) and true breeding values (B) of average daily body weight gain for 303 Ningxiang pigs.

Phenotypes of average daily body weight gain (ADG) were simulated for the 303 Ningxiang pigs (48 males and 255 females) with the GGP 50K SNP genotypes. Briefly, 2,000 SNPs, each with MAF > 0.10 were randomly assigned to be QTLs affecting ADG. The QTL additive genetic effects were simulated following a Gamma distribution, where the shape parameter was 0.4 and the scale parameter was 1.66 [32], and then re-centered at zero. The distribution of simulated QTL effects is shown in Fig 1A. Under the assumption of additive genetic inheritance, the phenotype of ADG for an animal, say animal *j* of sex *i*, was simulated as follows:

$$y_{ij}=\mu +{sex}_{i}+a_{j}+e_{ij}$$ (4)

where *a*_*j*_ was the animal’s true additive genetic value, *sex*_*i*_ was the sex effect, and *e*_*ij*_ was the residual term. The true breeding value of an individual was computed to be the sum of the simulated QTL effects for that animal. The distribution of true breeding values for the 303 Ningxiang pigs is shown in Fig 1B. The heritability of ADG was *h*^2^ = 0.28, mimicking the heritability estimate from a previous study [33]. The sex effects on ADG were 50 g for boars and 0 g for gilts, and the generation interval was set to be two years. For simplicity, the additive QTL variance explained all additive genetic variance, and the residual term was sampled from the distribution N ($0,\sigma_{e}^{2}=3461.7$). Then, breeding values of the 303 animals were estimated by BLUP using the same model as (4), where $a_{i}∼N(0,\sigma_{a}^{2}=1421.3)$ with *cov*(*a*_*i*_,*a*_*j*_) = 2*k*_*ij*_, and *k*_*ij*_ was the estimated genomic kinship coefficient between animals *i* and *j*.

Before the implementation of genomic OCS and genomic selection, SNP effects on EBV of ADG were estimated using ridge-regression BLUP [34] based on the following linear model:

$$u_{i}=\sum_{j=1}^{M}x_{ij}b_{j}+\epsilon_{j}$$ (5)

where *u*_*i*_ was the EBV of ADG of *i*-th individual, which was estimated using the model (4), *x*_*ij*_ was the genotype of the *j*-th SNP for the *i*-th individual, which was coded numerically as 0 (AA), 1 (AB), and 2 (BB) respectively, *b*_*j*_ was the association effects for the *j*-th SNP, and *ϵ*_*j*_ was an error term. Then, the genomic-estimated breeding value (GEBV) of each animal was taken to be the sum of estimated SNP effects corresponding to all the SNP genotypes that this animal had.

### Genomic OCS and genomic selection

In the present study, the 303 animals were treated as founders. Let $G=XX^{\prime }/\{2\sum_{j=1}^{K}p_{j}(1-p_{j})\}$ be the matrix of realized genomic relationships, where ***X*** is the centered SNP genotype matrix for all breeding candidates and $2\sum_{j=1}^{K}p_{j}(1-p_{j})$ is twice the sum of heterozygosities of the SNP markers [35], and ***c*** be a vector of proportional contribution of individuals to the next generation. Then, the inbreeding and coancestry for a given choice of *c* is defined as:

$$K(c,G|X)=\frac{1}{2}c^{\prime }Gc$$ (6)

Further, let $\hat{a}$ be the vector of GEBV for the trait. The genetic gain was taken to be the weighted average of expected breeding value of the progeny:

$$G(c,b)=c^{\prime \hat{a}}$$ (7)

In the above, the restrictions on the weights were *c*_*i*_≥0 for candidate *i* and $\sum_{i=1}^{N}c_{i}=1$, and *N* was the total number of candidates. For each sex, we had $\sum_{i=1}^{N_{s}}c_{i}=0.5$, and *N*_*s*_ was the number of animals of a specific sex. The objective was to maximize genetic gain, subject to the restriction that the maximum kinship was no greater than an upper bound of kinship, set up as follows:

$$K_{ub}=\bar{K}+(1-\bar{K})/(2N_{e}L)$$ (8)

where $\bar{K}$ was the average kinship, *N*_*e*_ was the effective population size, and *L* was the generation interval. The optimal ***c*** that maximized (7) was obtained by maximizing the following Lagrangian objective function [25]:

$$H=c^{\prime }b-\lambda_{0}(2K-c^{\prime }Gc)-\lambda (c^{\prime }Q-r)$$ (9)

where *λ*_0_ and ***λ*** were the Lagrangian multipliers (***λ*** was a vector of length 2), ***Q*** was a known incidence matrix for the sex of candidates, and ***r*** was a vector of 0.5’s of length 2. This problem can be solved either using Lagrangian multipliers [25] or using semidefinite programming [17]. Thus, genomic OCS can be viewed as a form of genomic selection with constraints on the rate of inbreeding. In contrast, the truncate genomic selection maximizes genetic gain without posing restrictions on inbreeding.

Long-term selection on simulated average daily gain was conducted for ten generations using genomic OCS and genomic selection, respectively, with the set of 303 Ningxiang pigs used as the base population. The purpose was to evaluate the utility of using genomic OCS as a sustainable selection strategy for Ningxiang pigs. For genomic selection, ten sires and 50 dams with the largest GEBV of ADG were selected and mated randomly in each generation. Genomic OCS was conducted under two scenarios. In the first scenario (denoted by GOCS_S), the top 10 sires with the largest contribution rates were selected, and each chosen sire was mated to 5 dams, which had the smallest kinships with this sire. This was to mimic the real situations in which the highly-intensive selection is not desirable, but minimizing kinships in parents (and hence inbreeding in progenies) is of more relevance. Thus, we limited selection for genetic gain on sires only. However, OCS requires constraining the average coancestry of selected parents, which otherwise is not met if each sire is randomly mated with equal probability to each unselected dam. Hence, minimizing kinships between selected parents in the present study was achieved by mating each chosen sire to five dams, which had the smallest kinships with this sire. In the second scenario (denoted by GOCS_SD), GOCS was implemented more closely following Sonesson et al. [14], in which sires and dams were selected with the constraint of inbreeding among selected parents. Then, each chosen sire was randomly mated to five chosen dams.

Progeny genotypes were simulated using a stochastic procedure, given the parental haplotypes. The SNP map was derived from the porcine genomic assembly 10.2 (Sscrofa10.2), and the gametes with recombination were produced by drawing a number of crossovers from a Poisson distribution with the mean being 1 per Morgan. The position of crossovers was taken at random without inference. A mutation rate of 1 × 10^−8^ per nucleotide was simulated for all chromosomes, regardless of if the locus is causative or non-causative. For each type of mating and selection, long term selection on simulated phenotypes was duplicated stochastically 100 times. Simulation and genomic OCS were conducted using an internal R package called GMT, developed at Neogen GeneSeek.

## Results and discussion

### Genomic inbreeding of Ningxiang pigs

**Fig 2:**
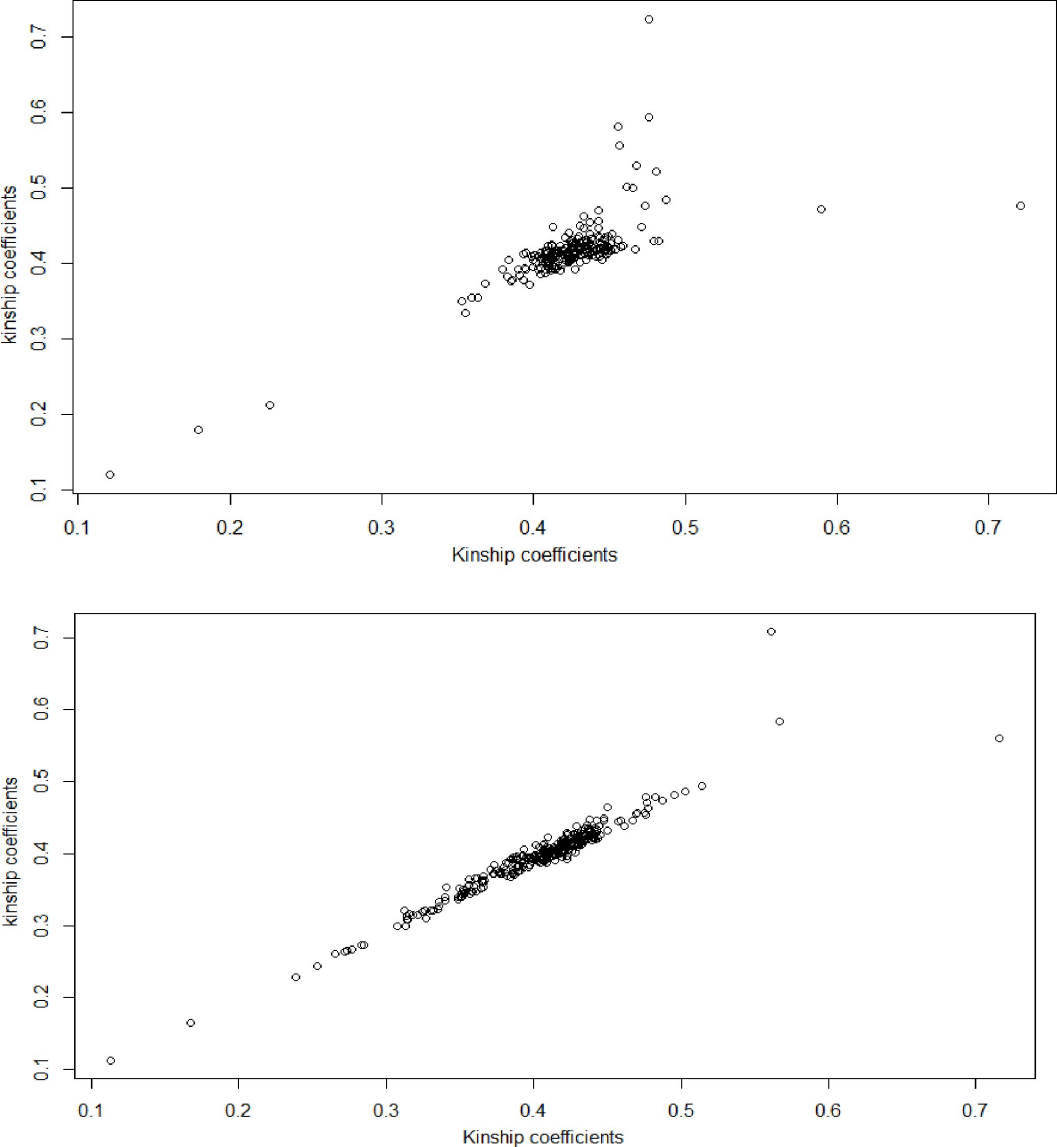
Correlation plots of genomic-estimated kinship coefficients between individual animals within each of two Ningxiang pig populations.

Average LW-inbreeding coefficients were 0.41 and 0.37 for the two sets of Ningxiang pigs genotyped with the Illumina PorcineSNP60 or the GGP Porcine 50K, respectively. The corresponding 95% confidence intervals were between 0.32 and 0.51 in NXP set 1, and between 0.25 and 0.46 in NXP set 2. The average LW-inbreeding coefficient in NXP set 1 was slightly larger than that in NXP set 2. There was evidence that, based on genomic-estimated kinship coefficients, animals in the first set were more closely related to each other than those in the second set (Fig 2). This difference could be due to actual differences in genetic diversity levels between groups, or sampling bias, or using different SNP arrays (Illumina Porcine60K for NXP set 1 vs. GGP Porcine 50K SNP for NXP set 2).

Average LW-inbreeding in these Ningxiang pigs was roughly comparable to the genetic diversity of two closed Iberian pig lines [36] but considerably smaller than inbreeding of Guizhou miniature pigs and Mam miniature pigs (0.59 and 0.48, respectively) [37]. It has been previously reported that animals in geographically isolated areas tend to have higher inbreeding due to the mating of closely related individuals [38,39].

Average ROH-inbreeding coefficients were estimated to be 0.24 and 0.25, respectively, in the two sets of Ningxiang pigs. The 95% confidence intervals of ROH-inbreeding were approximately between 0.16 and 0.35 in both datasets. Overall, ROH-inbreeding coefficients were smaller and more consistent between datasets than LW-inbreeding coefficients. It is generally acknowledged that ROH-inbreeding provides a more accurate measure of IBD than LW-inbreeding [40,41] because two alleles carried by an individual at a locus can be identical-by-state (IBS), inherited from two different ancestors, instead of being IBD from the same ancestor. Furthermore, as the length of ROH increases, the chance of being IBS for a chromosomal region drastically decreases. Thus, ROH-inbreeding coefficients vary with the length of ROH utilized. In the present study, ROH was defined for a minimum of 20 SNPs long.

Inbreeding is associated with a wide range of genetic phenomena, which include a decrease in genetic diversity of finite populations, a decrease in effective population size, genetic drift, changes in population structure, deviations from Hardy-Weinberg equilibrium, and decreases in population means [42]. Often, inbreeding leads to inbreeding depression. For marker loci to indicate inbreeding depression, their heterozygosity should be correlated with the heterozygosity of functional genes affecting fitness [43]. There are two hypotheses under the broad category of heterozygosity-fitness correlation (HFC) theory for the explanation of inbreeding depression. Firstly, the “general effect hypothesis” assumes that multilocus heterozygosity (MLH) reflects genomic heterozygosity, and the association emerges because variation in inbreeding causes heterozygosity to be correlated across loci. This phenomenon was termed identity disequilibrium by Weir & Cockerham [44]. Secondly, the “local effect hypothesis” states that one or a few of the markers are in linkage disequilibrium (LD) with a trait locus under balancing selection, creating a pattern whereby heterozygosity at the gene and marker are correlated [43,45]. Both mechanisms can, therefore, be united under an inbreeding or general effect model [43]. In the present study, Ningxiang pigs showed considerably increased inbreeding, as compared to developed pig breeds or lines. Thus, our results emphasize that it is highly important to control the rate of inbreeding in the preservation and genetic improvement programs of Ningxiang pigs. An evaluation of possible inbreeding depression in Ningxiang pigs was not conducted in the present study, but it remains a subject of interest for future studies.

### Comparison of GOCS and GS

**Fig 3:**
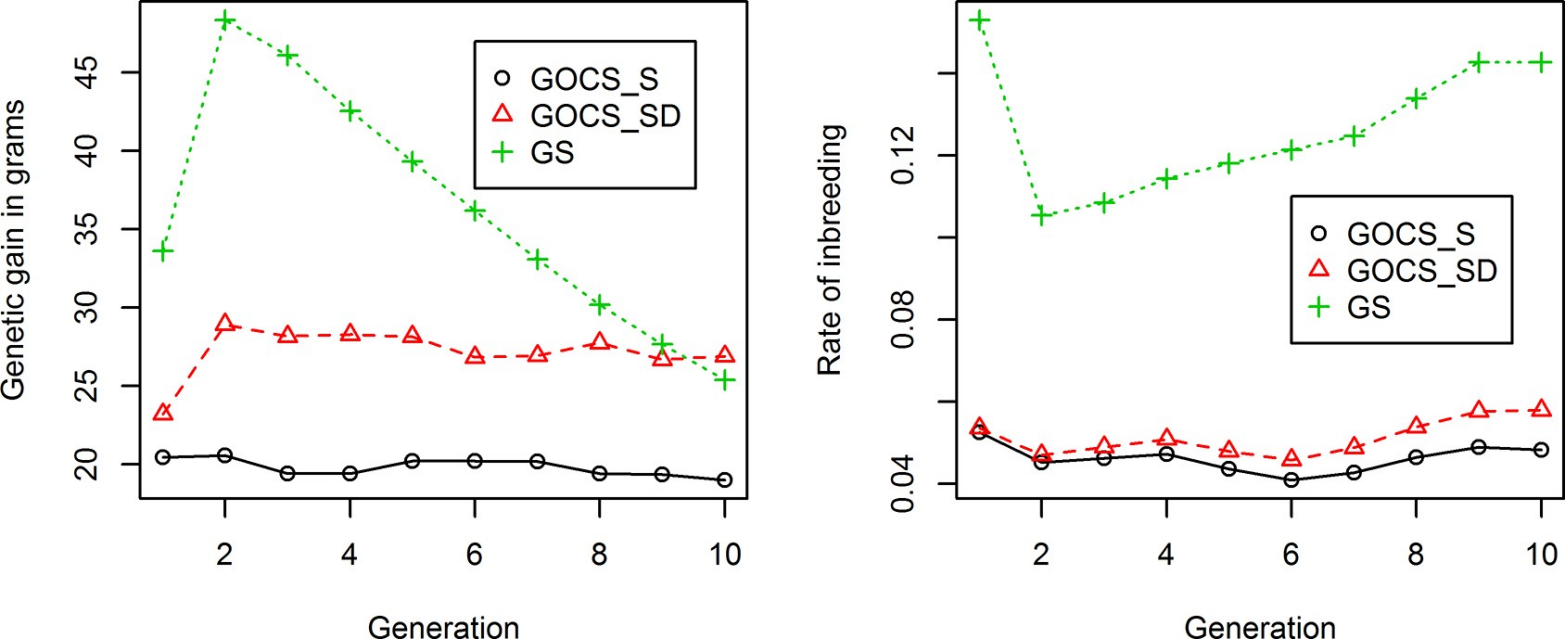
Comparison of simulated genetic gain on average daily body weight gain (A) and average inbreeding based on runs of homozygosity (B), which were obtained under two scenarios of genomic OCS (GOCS_S and GOCS_SD) and genomic selection (GS) without inbreeding control, respectively.

In the NXP set 2, genomic selection led to a high rate of genetic gain in ADG in the first two generations (Fig 3A). Conversely, the rate of ROH-inbreeding dropped in the second generation and then steadily increased through the tenth generation. The average rate of ROH-inbreeding was larger than 10% at each of the ten generations of selection (Fig 3B). The rate of inbreeding was slightly higher at generation one than the remaining generations because there was a small number of candidate sires (i.e., 48 sires) to select. The genetic gain was maximum in the second generation, and it began to decrease after that (Fig 3A). In general, genetic gain tended to decrease from generation 2 to 10. The genetic gain in generation one was not the largest because the “founder” population size was small. The decrease of population means in the truncated genomic selection is a consequence of drastically reduced genetic variance due to inbreeding. In reality, this can worsen by inbreeding depression, due to the presence of dominance and overdominance inheritance, in which certain recessive alleles have negative effects or are associated with reduced fitness. The inbreeding depression effect is not the same for fitness-related traits and production traits, but the reduction in the performance of the population can follow the same principle. The present study, however, did not consider dominant and over-dominant effects.

In the two scenarios of genomic OCS, the rate of inbreeding per generation was controlled to be less than 5% for each of the ten generations of selection under scenario 1 (Fig 3B), and mostly less than 5% for all the generations except for the first and last generations under scenario 2 (Fig 3B). The rate of inbreeding was similar between the two scenarios of GOCS, yet selecting on both sires and dams led to a considerably larger genetic gain and a slightly larger rate of inbreeding than selecting on sires only. While the rate of genetic gain per generation obtained using GS was substantially larger than those obtained by the two scenarios of genomic OCS in the beginning generations of selection, the difference in genetic gain between GS and Genomic OCS reduced quickly at latter generations (Fig 3A). At generation ten, the difference in the realized rates of genetic gain between GS and GOCS_SD diminished and ended up with even a slightly higher genetic gain with GOCS_SD (Fig 3A). This could be due to the greater loss of genetic variance with GS as inbreeding accumulated and changes in linkage disequilibrium occurred between QTL and linked markers. Also, some QTLs were fixed at generation ten. Therefore, Genomic OCS had at least a comparable genetic gain yet had a much lower risk of increasing breeding (and reducing genetic diversity) than GS without controlling inbreeding.

Arguably, genomic OCS is becoming a well-established method that can be used in many practical animal breeding applications. Nevertheless, improvements are also emerging. In genomic OCS, a measure of genetic gain is often taken to be the total expected breeding value of the progeny. Still, it carries no information about the variability of breeding values among full-sibs. Akdemir and Sanchez [46] introduced a measure called the risk of a mating plan, such that the expected merit of the progeny was increased by a small amount proportional to their expected variance (standard deviation) computed under the assumption of infinitesimal genetic effects, with the extent of increment controlled by a scalar parameter. Likewise, avoiding the loss of rare favorable alleles has also received attention when increasing long-term selection gain. Thus, Jannink [47] proposed a weighted strategy of genomic selection (WGS), in which the effect of rare favorable alleles is amplified following theory by Goddard [48]. Likewise, De Beukelaer et al. [49] proposed two new sets of selection methods that maximize a weighted index balancing genetic gain with controlling expected heterozygosity or maintaining rare alleles, which can have added value to GOCS.

## Conclusion

Genomic OCS provides a well-established approach to balancing genetic gain and the loss of genetic diversity. The present results investigated the performance of GOCS in Ningxiang pigs with observed genotypes and simulated phenotypes of average daily body weight gain. Two scenarios of genomic OCS were examined, in comparison with GS without controlling inbreeding. Scenario No. 1 (GOCS_S) presented a modified version of genomic OCS for indigenous breeds such as Ningxiang pigs, in which selection for genetic gain operated on sires only but constraining kinships on both sires and dams. This type of non-random mating arrangement was a necessary step with scenario No. 1. In genomic OCS under scenario No. 2 (GOCS_SD), both sires and dams with the largest contribution rates were selected, and the kinships between selected parents were constrained. The present results supported the utility of genomic OCS for the breeding of Ningxiang pigs because it can maintain a sustainable long-term genetic gain while controlling the rate of inbreeding within a desirable range for most of the generations of selection. The present study involved only a single trait, namely, average daily gain. In a real situation, multiple traits of economic importance will need to be considered, which could include, for example, growth rate, intramuscular fat, and reproductive performance. Meanwhile, future considerations may also include investigating alternative, more cost-effective forms of genomic mating or genomic OCS for sustainable genetic improvement of local breeds [46].
